# Supplementary material for: Complexome profiling of the Chlamydomonas psb28 mutant reveals TEF5 as an early PSII assembly factor
Source: Plant Cell. 2025 Mar 17;37(6):koaf055. doi: 10.1093/plcell/koaf055 (PMC12164590; doi:10.1093/plcell/koaf055)
Supplement: koaf055_Supplementary_Data [file koaf055_supplementary_data.zip › psb28_interactive.zip!/plots/intensity/qualityPlots/peptides/COX2B_Cre01.g049500.t1.2.html]

### Cre01.g049500.t1.2

Trivial name: COX2B  
SeqCoverage [median]: 60.10  
Mass: 17.25 kDa  
  
 

| Sequence | MissedCleavage | Mass [Da] | wtIntensity | muIntensity | Score |
| --- | --- | --- | --- | --- | --- |
| LKADPSFR | 1 | 932.51 | 7032.08 | 5892.45 | 143.00 |
| LKADPSFRAELKDR | 3 | 1644.89 | 1029.17 | 12523.38 | 74.79 |
| LNQVWMSINR | 0 | 1259.64 | 17812.21 | 21099.71 | 190.62 |
| LVLPTNTLIR | 0 | 1138.71 | 181630.72 | 179194.74 | 149.65 |
| MDAVPGR | 0 | 744.36 | 6399.76 | 5281.46 | 97.07 |
| QFLTEYVK | 0 | 1026.54 | 13671.37 | 8222.37 | 149.40 |
| QFLTEYVKK | 1 | 1154.63 | 18680.79 | 25363.19 | 131.52 |
| SESKDQLK | 1 | 933.48 | 1756.67 | 1572.75 | 103.56 |
| SESKDQLKEK | 2 | 1190.61 | 116579.21 | 149253.15 | 206.97 |
| VLEVDER | 0 | 858.44 | 145830.05 | 155635.36 | 138.85 |
| VLEVDERLVLPTNTLIR | 1 | 1979.14 | 1875.60 | 5480.91 | 68.94 |
| VPASVPISYNFDSYMLTEVQPGQLR | 0 | 2810.39 | 7933.12 | 0.00 | 122.18 |
|  |  |  | 520230.7 | 569519.5 |  |
